# Extreme Paralysis Following Rocuronium Administration in a Myasthenia Gravis Patient: A Case Report

**DOI:** 10.5811/cpcem.1548

**Published:** 2023-07-19

**Authors:** Kelsey Billups, Charlotte Collins, Aimee Weber

**Affiliations:** * Medical University of South Carolina, Department of Pharmacy, Charleston, South Carolina; † Medical University of South Carolina, Department of Emergency Medicine, Charleston, South Carolina; ‡ Medical University of South Carolina, Department of Neurosurgery, Charleston, South Carolina

**Keywords:** case report, rapid sequence intubation, myasthenia gravis, rocuronium, paralytic

## Abstract

**Introduction:**

The use of paralytics during rapid sequence intubation (RSI) in patients with myasthenia gravis (MG) remains a controversial topic in emergency medicine. Due to fewer functional acetylcholine receptors, these patients can be both sensitive and resistant to different types of neuromuscular blocking agents (NMBA). Their atypical sensitivity to non-depolarizing NMBAs such as rocuronium can increase both the duration and depth of paralysis after its use at typical RSI doses. However, the extent of rocuronium’s prolonged duration of effect in patients with MG has yet to be quantified in an emergency department setting.

**Case Report:**

We describe a case wherein a full RSI dose of 1.2 milligrams per kilogram of rocuronium led to a prolonged 232-minute duration of paralysis in a patient with MG. This sustained paralysis was suspected but only confirmed after the patient received the reversal agent sugammadex. Once administered, an acute change in neurologic function was seen, and the patient was emergently taken to the operating room for neurosurgical intervention.

**Conclusion:**

When intubating patients with MG, many emergency physicians are aware that using paralytics during RSI provides several challenges. If not properly dose-reduced, rocuronium may exert its paralytic effects for up to four hours in patients with MG. This unique case highlights the importance of personalizing care for this patient population before, during, and after RSI.

## INTRODUCTION

In patients with myasthenia gravis (MG), the decision of which neuromuscular blocking agent (NMBA) to use during rapid sequence intubation (RSI), if any, is often controversial due to the atypical and unpredictable response to both succinylcholine and rocuronium.^1^,^2^ Patients with MG have fewer functional acetylcholine receptors compared to other patients. Therefore, a decreased sensitivity to the depolarizing NMBA succinylcholine is seen.^3^ Adult patients may require up to 2.6 times the normal induction dose for appropriate muscular relaxation. Contrastingly, patients with MG have an increased response to non-depolarizing NMBAs such as rocuronium.

Literature suggests that a dose reduction of 50–90% may still provide adequate neuromuscular blockade.^1^,^4^ This increase in sensitivity can impact the depth and duration of paralysis. However, compared to other paralytics, the actual extent of these effects is not well known or reported with rocuronium use.^5^ A one-time administration of 0.6–1.2 milligrams per kilogram (mg/kg) of rocuronium during RSI has a 37–72 minute duration of action in patients without MG.^6^,^7^ In the MG population, the expected length of effect after RSI with a full or reduced dose in the emergency department (ED) is unknown.

The uncertainty of rocuronium’s maximum duration of action is likely due to prompt, continuous sedation following RSI and rare need for extubating patients with MG in the ED. Additionally, all previously reported literature reporting the duration of paralysis solely reflects the length of the associated surgery and subsequent extubation.^1^,^4^,^8^,^9^ Due to this gap in knowledge, emergency physicians are likely unaware of how long their patients may be paralyzed following RSI with rocuronium and how critical it is to reduce the dose when using this NMBA for intubating patients with MG. We report a case in which an ED patient with MG required sugammadex administration for the reversal of rocuronium nearly four hours after administration. This case report highlights the special considerations that must be taken when caring for these patients before and after endotracheal intubation.

## CASE REPORT

A 66-year-old patient with a history of MG, chronic lymphocytic leukemia, diabetes, hypertension, and thyroid disease presented to the ED with altered mental status and fevers after falling. Upon arrival, vitals were taken, and the emergency physician performed the initial physical exam. The patient was noted to be obtunded but responsive to painful stimuli. Other than stating his name when asked, the patient was unable to follow commands. His pupils were equal and reactive bilaterally without ocular nystagmus. Given his continued decline in mental and respiratory status, the patient (estimated weight of 97.7 kg) was intubated in the ED with etomidate 0.3 mg/kg and a full RSI dose of rocuronium 1.2 mg/kg. A propofol infusion was initiated at 20 micrograms/kg/minute, and a computed tomography (CT) head was ordered. The patient’s heart rate (HR) and blood pressure (BP) at that time were 117 beats per minute (BPM) and 140/79 millimeters of mercury (mm Hg), respectively. (See Table for summary of patient’s ED course.)

Two hours after RSI, the emergency physician was made aware of the following CT head results: acute right subdural hematoma (SDH) measuring 1.5 centimeters with 12 mm shift, downward transtentorial herniation, subfalcine herniation, and uncal herniation. Shortly thereafter, neurosurgery was consulted for neurosurgical evaluation. Approximately three hours after RSI the patient’s continuous sedation was stopped, and a bedside examination was performed by the neurosurgery team 20 minutes later. Prior to propofol discontinuation, the patient’s HR was 100 BPM, and BP was 95/57 mm Hg. The patient had no cough and was not opening his eyes. his pupils were bilaterally reactive on examination.

The neurosurgeon then performed a bedside train of four (TOF) to assess the patient’s level of paralysis. Train of four is a peripheral nerve stimulation used as a qualitative assessment of neuromuscular blockade. Electrical current is applied to the peripheral nerve, and the reactive muscular twitches are visually assessed. Train of four can be done at the ulnar or facial nerve. The test sends four impulses with an output between 10–70 milliamperes (mA). If a patient twitches 4/4 times, the patient is considered not paralyzed. If a patient twitches 0/4 times, the patient is fully paralyzed. Two of four twitches at maximum output would indicate partial paralysis.

**CPC-EM Capsule**

What do we already know about this clinical entity?

*Emergency medicine physicians are aware patients with myasthenia gravis have an atypical response to paralytics when used during rapid sequence intubation.*

What makes this presentation of disease reportable?

*This case quantifies the longest duration of paralysis ever reported when using rocuronium for rapid sequence intubation in a myasthenia gravis patient.*

What is the major learning point?

*Patients with myasthenia gravis must receive personalized care when intubated with paralytics, especially rocuronium, in the emergency department.*

How might this improve emergency medicine practice?

*Physicians in the Emergency Department are now better equipped in managing patients with myasthenia gravis during and after rapid sequence intubation.*

His TOF was 0/4 in each upper extremity. The patient did have one slight facial twitch with the stimulator maximized to an output of 70 mA, indicating near-complete neuromuscular blockade. At that time, sugammadex was ordered to determine whether the patient’s examination was due to prolonged paralysis from rocuronium or his acute SDH.

Nearly four hours (232 minutes) after 1.2 mg/kg of rocuronium was given, sugammadex 4 mg/kg was administered. A final physical examination was immediately performed by the same neurosurgeon. The patient was found to have a motor exam positive for bilateral upper extremity flexion and bilateral lower extremity withdrawal and flexion. After these findings, the patient was then taken to the operating room (OR) for right-sided craniectomy and evacuation of their SDH. Thirty minutes following sugammadex administration, the patient’s HR and BP were 90 BPM and 77/39 mm Hg, respectively.

## DISCUSSION

This case report describes the longest duration of paralysis following rocuronium administration in the ED for RSI in a patient with MG. To assess neurologic function, our patient required sugammadex for reversal of rocuronium 232 minutes after administration. The change in physical examination after sugammadex administration, indicating excessive prolonged paralysis, highlights the challenges of using and dosing rocuronium appropriately in this patient population. Our patient’s clinical course and disposition drastically changed after sugammadex was given. Currently, literature describing rocuronium use followed by reversal with sugammadex in adult patients with MG is in the OR setting only.^1^,^4^,^8^–^10^ We believe this case report gives emergency physicians unique guidance and reasoning into managing this patient population peri-endotracheal intubation. It may also help explain any unexpected neurologic changes, or lack thereof, seen when managing these patients post-intubation.

Patients with MG have autoimmune destruction of nicotinic acetylcholine receptors causing an unpredictable hyper-response to the non-depolarizing NMBA rocuronium. The effective dose varies between patients and has been reported to range between 0.15 and 1.2 mg/kg. Fujimoto and colleagues attempted to identify factors that would put MG patients at risk of an increased response to rocuronium. They found that patients with a lower baseline TOF and younger age of MG diagnosis achieved adequate paralysis in the OR with 0.15 mg/kg of rocuronium.^8^ Unfortunately, as commonly seen in the ED, neither of these factors were known in our patient given the emergent need for intubation. While some patients with MG achieve paralysis with a reduced dose of rocuronium (0.15–0.3 mg/kg), others may require and safely respond to a dose of 1–1.2 mg/kg.^4^,^8^–^10^

Our patient was given 1.2 mg/kg of rocuronium to ensure immediate, adequate paralysis for emergent RSI. Prior to the current case report, the longest reported duration of paralysis after rocuronium intravenous (IV) push ranged upward to 120 and 180 minutes, using a full and reduced RSI dose, respectively.^4^,^8^ However, these times reflect the duration of the elective procedure performed in the OR, not rocuronium’s full duration of effect. Unlike the current case report, those cases do not provide an accurate description of how long rocuronium’s paralysis may realistically last after RSI in the ED.

Despite rocuronium having an unpredictable effect in patients with MG, the ability of sugammadex to reverse this paralytic may offset this unwanted pharmacologic property. Sugammadex, first used for rocuronium reversal in a MG patient in 2010, binds to and reverses the effects of non-depolarizing NMBAs such as rocuronium and vecuronium.^7^,^11^ This medication has a quick reversal time of less than five minutes and does not carry a risk of inducing a myasthenic crisis.^12^ Sugammadex dosing seems to be consistent between patients with and without MG.

Dosing is determined by depth of neuromuscular blockade based on TOF, dose of rocuronium used, and elapsed time from non-depolarizing NMBA administration.^7^ A typical dose of 1.25–4 mg/kg should obtain sufficient reversal of rocuronium in patients with MG after moderate to deep neuromuscular blockade.^1^,^12^,^13^ A dose of 16 mg/kg should be reserved for rapid reversal immediately after rocuronium administration.^13^ Adverse reactions include bradycardia, hypotension, anaphylactic reactions, and urticaria.^14^ No bradycardia was seen in our patient. However, hypotension did occur as their BP declined from 95/57 mm Hg to 77/39 mm Hg after sugammadex administration. This reduction was self-limiting, and no interventions were required.

Succinylcholine is an alternative NMBA that may be used for RSI in patients with MG. However, the use of this depolarizing paralytic comes with its own challenges. Since myasthenic patients have fewer functioning acetylcholine receptors, succinylcholine resistance is commonly seen. Rather than 1.5 mg/kg, a dose up to 2 mg/kg may be necessary for adequate intubation conditions.^3^ Additionally, maintenance medications for MG such as pyridostigmine and rivastigmine may prolong the neuromuscular blocking effects of succinylcholine, as they inhibit an enzyme responsible for metabolizing this paralytic.^5^ Due to the innate challenges of using either rocuronium or succinylcholine for RSI in patients with MG, some physicians may choose to withhold NMBAs entirely. It’s reasonable to consider induction using IV sedation, analgesia, and/or topical anesthesia without a NMBA in these patients.^2^ However, this approach has been most successfully reported in the perioperative setting.^15^

## CONCLUSION

This case describes the longest reported duration of paralysis after rocuronium administration in a patient with MG requiring emergent intubation in the ED. Nearly four hours after receiving a full induction dose of 1.2 mg/kg, our patient required sugammadex to reverse the effects of rocuronium. Many emergency physicians are aware that using paralytics during RSI in this patient population provides several challenges. This case report quantifies the extent of rocuronium sensitivity in patients with MG. Furthermore, it provides the emergency physician better insight into the duration of paralysis, the importance of rocuronium dose reduction, and the impact these clinical decisions can have on their patient’s post-intubation medical care.

## Figures and Tables

**Table:** Summary of Emergency Department Course.

	13:05–13:15	15:40–15:45	18:35–18:55	19:31–19:33	19:44–19:50
Event	Arrival to ED Physical exam #1	Rapid sequence intubation	Physical exam #2	Physical exam #3	Decision made to pursue OR
Medication administration		Etomidate 30 mg (0.3 mg/kg) Rocuronium 120 mg (1.2 mg/kg) Propofol started at 20 mcg/kg/min	Propofol stopped	Sugammadex 400 mg (4 mg/kg)	
Vitals	BP 168/80 mmHg HR 95 bpm RR 24 per min Temperature 39.4C (oral) Oxygen saturation 96%	BP 140/79 mmHg HR 117 bpm RR 14 per min			BP 77/39–81/53 mmHg

*ED*, emergency department; *OR*, operating room; *mg/kg*, milligrams per kilogram; *mcg*, microgram; *BP*, blood pressure; *mm Hg*, millimeters of mercury; *HR*, heart rate; *bpm*, beats per minute; *RR*, respiration rate.
